# Three-dimensional evaluation of maxillary tooth movement in extraction patients with three different miniscrew anchorage systems: a randomized controlled trial

**DOI:** 10.1186/s40510-022-00441-4

**Published:** 2022-12-19

**Authors:** Liwen Zhang, Runzhi Guo, Baohua Xu, Yue Wang, Weiran Li

**Affiliations:** 1 Department of Dental Medical Center, China-Japan Friendship Hospital, Beijing, People’s Republic of China; 2 Department of Orthodontics, Peking University School and Hospital of Stomatology, 22 Zhongguancun Avenue South, Haidian District, Beijing, 100081 People’s Republic of China

**Keywords:** Maxillary tooth movement, Miniscrew, Cone beam computed tomography, Randomized controlled trial

## Abstract

**Objective:**

To compare the three-dimensional (3-D) movement of maxillary teeth in response to three common miniscrew anchorage systems in extraction patients with maxillary dentoalveolar protrusion.

**Materials and methods:**

The study employed a randomized controlled single-blinded design with three arms. Thirty extraction patients who required maximum anchorage to retract maxillary anterior teeth were included and randomly allocated into three treatment groups: space closure with direct miniscrew anchorage and low crimpable hooks (DL group), indirect miniscrew anchorage and low crimpable hooks (IL group), and direct miniscrew anchorage and high crimpable hooks (DH group). Cone beam computed tomography (CBCT) images of all included patients were obtained immediately before (T0) and after (T1) space closure. The outcomes were 3-D positional changes of maxillary central incisor, lateral incisor, canine, second premolar, and first molar. The repeated measures analysis of variance with post hoc LSD test was used to evaluate differences among groups.

**Results:**

A significant intrusion (− 1.34 mm; 95% CI, − 1.60 mm, 1.08 mm) and buccal (− 6.92°; 95% CI, − 8.67°, − 5.13°) and distal (4.90°; 95% CI, 3.75°, 6.04°) inclination of the maxillary first molars were observed in the DL group, compared to the other two groups. The mesial movement (− 0.40 mm; 95% CI, − 0.83 mm, − 0.03 mm) of the maxillary first molars was found in the IL group, while the DL (0.44 mm; 95% CI, 0.15 mm, 0.73 mm) and IL (0.62 mm; 95% CI, 0.28 mm, 0.96 mm) groups exhibited distal movement. In the DH group, the lingual inclination changes of maxillary central incisor (5.04°; 95% CI, 2.82°, 7.26°) were significantly lower, which is indicative of good lingual root torque control of the maxillary anterior teeth.

**Conclusion:**

Three miniscrew anchorage systems produced significantly different 3-D maxillary tooth movement. The maxillary first molars were significantly buccally and distally inclined and intruded in patients using direct miniscrew anchorages with low crimpable hooks. Direct miniscrew anchorages with high crimpable hooks could help to achieve better lingual root torque control of the maxillary incisors.

*Trial registration* The trial was registered at www.chictr.org.cn (ChiCTR1900026960). Registered 27 October 2019.

## Introduction

Maxillary dentoalveolar protrusion characterized by proclined upper incisor and protruding lips is common in Class I and Class II malocclusion. The treatment plan for this malocclusion often includes extraction of the maxillary first premolars to retract the anterior teeth and improve the facial profile [1]. During treatment, anchorage control is essential for maximizing the retraction of maxillary anterior tooth. Compared with traditional anchorage devices, miniscrews are more efficient and more comfortable; they can reduce the need of patient compliance [2, 3]. For maxillary protrusion patients, the use of miniscrews for orthodontic anchorage has become a routine treatment option [4]. During en masse retraction, miniscrews can be used either directly or indirectly [5]. Direct anchorage refers to the miniscrews being directly loaded to retract the anterior teeth, while indirect anchorage refers to the miniscrew being used indirectly to stabilize the posterior teeth, and then using these stabilized posterior teeth for anterior teeth retraction.

To achieve ideal occlusion, maxillary tooth movement during en masse retraction should be clearly characterized. Previous studies have compared the effects of conventional anchorage and direct miniscrew anchorage on maxillary tooth movement in maxillary protrusion patients [6–8]. Patients utilizing direct miniscrew anchorage exhibited significant intrusion of the maxillary anterior teeth and distal inclination of the maxillary first molars when compared to conventional anchorage. Because of directional differences in retraction forces, the sliding mechanics differ between direct and indirect miniscrew anchorage systems. In addition, the height of the anterior crimpable hooks can influence the retraction biomechanical paradigm, thereby affecting the pattern of maxillary tooth movement [2, 9]. A finite element study found that an increased anterior crimpable hook height could help decrease lingual tipping of the maxillary anterior teeth during en masse retraction [9]. Besides, most recent studies have used cephalometric tracing and digital dental model superimposition to evaluate orthodontic tooth movement [7, 10–12]. However, cephalometric images are two-dimensional and digital dental models can only evaluate crown movement; they cannot evaluate root movement. Compared with cephalometric and digital dental model analysis, cone beam computed tomography (CBCT) can accurately analyze tooth movement in three dimensions, including both crown and root movement. In addition, the accuracy and reliability of voxel-based superimposition based on the maxillary region have been previously validated [13, 14].

To our knowledge, the three-dimensional (3-D) movement of maxillary teeth has not been compared among miniscrew anchorage systems. Information regarding these differences will help orthodontists to choose the best miniscrew anchorage system for their patients, with the goal of avoiding unexpected tooth movement. Therefore, this study used CBCT to compare the 3-D movement of maxillary teeth among three common miniscrew anchorage systems.

## Materials and methods

### Study design

This three-arm randomized, controlled, single-blind clinical trial was conducted in accordance with the guidelines of the Consolidated Standards of Reporting Trials (CONSORT) [15]. The study protocol was approved by the ethics committee of the China-Japan Friendship Hospital (No. 2018–101–k73) and registered at the Chinese Clinical Trial Registry.

### Patient selection and setting

The recruitment of patients was conducted at the Department of Dental Medical Center of China-Japan Friendship Hospital. Patients were included if they met the following criteria: age 18–30 years; maxillary dentoalveolar protrusion with a Class I or Class II division I molar relationship; maxillary mild crowding (i.e., < 3 mm); a treatment plan that involved extraction of four premolars (four first premolars, or upper first and lower second premolars) and requiring retraction of the maxillary anterior teeth with maximum anchorage; healthy periodontal and temporomandibular statuses; no crowns or implants; and no diabetes or other systemic diseases. All patients provided written informed consent prior to enrollment in the study.

### Sample size calculation

Sample size calculation was performed using Power Analysis and Sample Size for Windows software (PASS 2000, NCSS, Kaysville, UT, USA). Considering that no previous study has analyzed the 3-D positions of the maxillary dentition, the sample size was calculated assuming that the vertical displacement of maxillary first molars was different among groups. Based on the findings of our preliminary study, 10 subjects per group were required to detect an absolute difference of 0.82 mm with 80% power at 5% significance level (two-sided). In our study, the left and right sides of the upper dentition in each patient were analyzed separately to increase the sample size. To allow for possible dropouts during the study, we enrolled 36 patients in total.

### Randomization, allocation concealment, and blinding

Using a simple 1:1:1 randomization procedure, the patients were randomly assigned into three equal groups: direct miniscrew anchorage with low crimpable hooks (DL), indirect miniscrew anchorage with low crimpable hooks (IL), and direct miniscrew anchorage with high crimpable hooks (DH). Randomization was performed using opaque envelopes that contained allocation sequences; the sequences were generated using Microsoft Excel 2013 (Microsoft Corporation, Redmond, WA, USA). Opaque, sealed, and sequentially numbered envelopes were used to perform the allocation concealment. The randomization and allocation concealment were implemented by an academic assistant who was not involved in the study. Considering the nature of the intervention, patients and investigators could not be blinded during the study. Hence, only the measurement was blinded, in accordance with a single-blind trial design. The CBCT images of all included patients were firstly coded by an examiner (YW) without showing patients’ names and group allocations. Another examiner (RZG) who was unaware of group assignments performed all of the measurements.

### Intervention

**Fig. 1:**
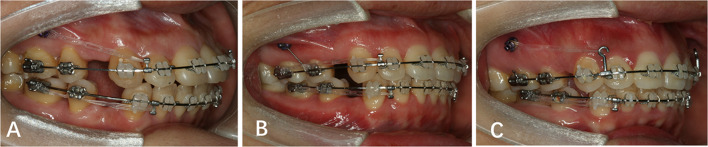
Illustration of three miniscrew anchorage systems: **A** Direct miniscrew anchorages with low crimpable hooks (DL group); **B** indirect miniscrew anchorages with low crimpable hooks (IL group); **C** direct miniscrew anchorages with high crimpable hooks (DH group)

All patients were treated with pre-adjusted MBT appliances using 0.022″ × 0.028″ slot brackets (3 M Unitek, Monrovia, CA, USA). To reduce sliding friction, the second molars were not bonded until extraction space closure [16]. After the initial alignment, miniscrews 1.7 mm in diameter and 8.0 mm in length (Ortho Easy, Forestadent, Pforzheim, Germany) were placed in the interradicular area between the second premolars and maxillary first molars, at 8 mm above the arch wire; all procedures were performed by the same operator. Three weeks after miniscrew insertion, the 0.019″ × 0.025″ stainless steel arch wires were used for en masse retraction of anterior teeth. As shown in Fig. 1, in the DL group, low crimpable hooks 2 mm in height (Shinye, Hangzhou, China) were placed between the lateral incisors and canines. Elastic chains were stretched from the miniscrews to the low crimpable hooks for direct anchorage. In the IL group, low crimpable hooks were used; miniscrews were tightened to the second premolar bracket with a 0.25-mm stainless steel ligature wire for indirect anchorage. The elastic chains were then stretched from the first molar tubes to the low crimpable hooks. In the DH group, high crimpable hooks 8 mm in height (Shinye, Hangzhou, China) were placed between the lateral incisors and canines. The elastic chains were stretched from the miniscrews to the high crimpable hooks for direct anchorage. In all three groups, a retraction force of 150 gf was applied and the elastic chains were replaced at 1-month intervals. To avoid confounding factors, intermaxillary elastics or additional torque control were not performed during the space closure.

### CBCT protocol and analysis

**Fig. 2:**
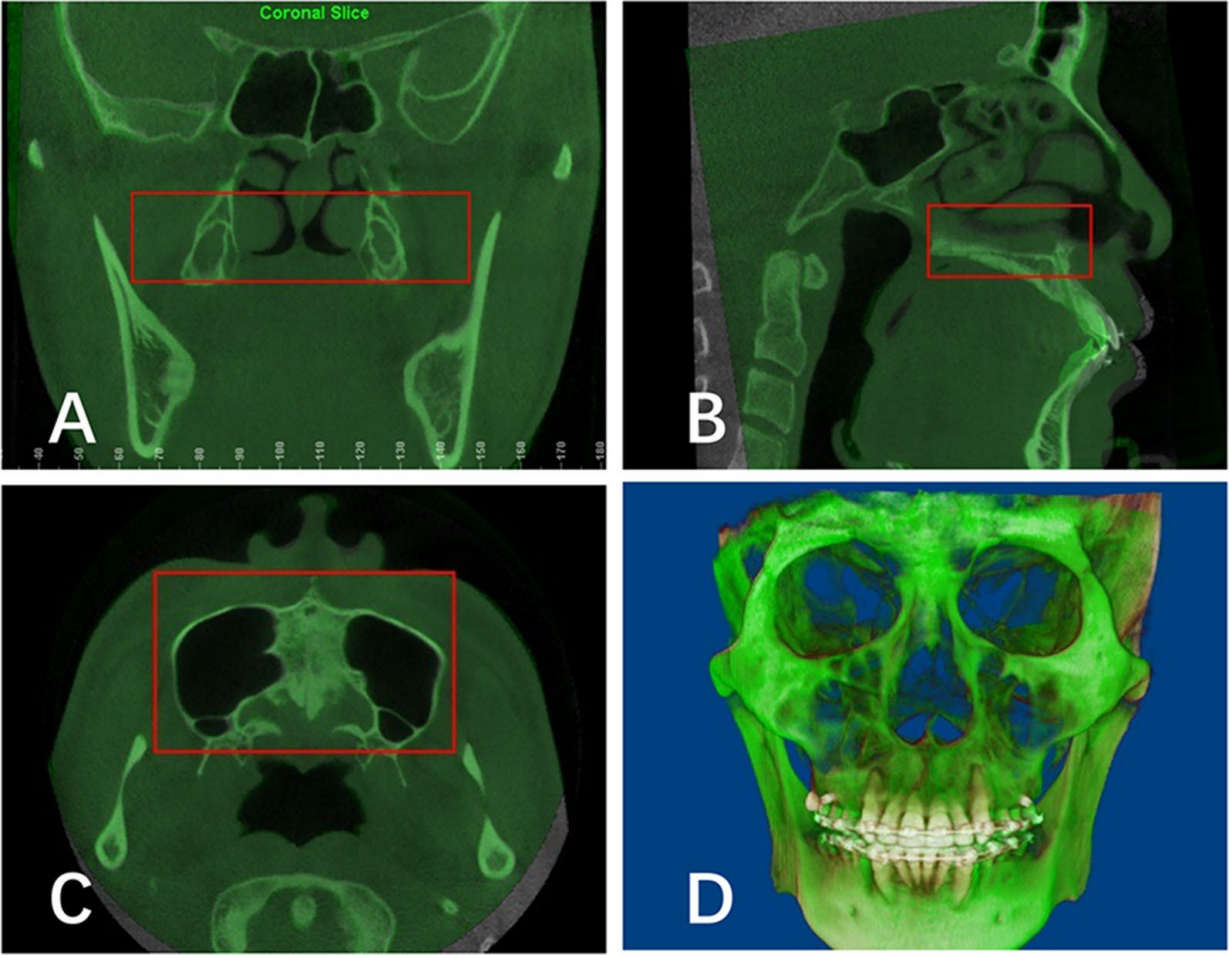
Voxel-based superimposition of pre-retraction and post-retraction CBCT based on the maxillary region. **A** The coronal, **B** sagittal, **C** axial and **D** 3-D frontal view of superimposition

In this study, the CBCT images of all included patients were acquired before (T0) and after (T1) space closure, with an interval of at least six months. The CBCT images were taken with 0.3 mm^3^ voxel size, 160 mm × 220 mm field of view, 110 kV tube voltage, 2.81 mA tube current and 3.6 s scan time, in accordance with a low-dose protocol [17]. The thyroid lead shielding was used to protect patients. These data were exported into DICOM format and further analyzed in Dolphin 3D software (ver. 11.7, Dolphin Imaging & Management Solutions, Chatsworth, CA, USA). The head orientation in all CBCT scans was standardized via manual identification. The Frankfort horizontal plane (Or-Po) was oriented as the horizontal plane; the plane connecting the nasion, anterior nasal spine, and posterior nasal spine (Na-ANS-PNS) was oriented as the midsagittal plane. After 3-D orientation, voxel-based superimposition of the CBCT images at T0 and T1 was performed with Dolphin 3D software. The maxillary region was selected for voxel-based superimposition as previously described [13, 14]. As shown in Fig. 2, the software matched the selected voxels, then automatically superimposed the T0 and T1 images.

A 3-D system of coordinates was established in the superimposition model to orient the landmarks. The Na-ANS-PNS plane was selected as the midsagittal plane (*X* plane) and the Frankfort horizontal plane was selected as the horizontal plane (*Y* plane); the coronal plane (*Z* plane) was oriented perpendicular to the midsagittal and horizontal planes. The PNS point was selected as the origin (0, 0, 0). Ten landmarks, with coordinates in (*x*, *y*, *z*) format, were manually positioned on each CBCT image. The definitions of the landmarks are listed in Table 1. The line connecting the crown and root landmarks represents the long axis of the maxillary teeth. The 3-D landmark coordinates were exported into Microsoft Excel 2013 and further evaluated using MathType software (ver. 5.0, Design Science, Long Beach, CA, USA).

**Table 1:** Definitions of the landmarks in the study

Landmark	Definition
U6c	Mesiobuccal cusp tip of the maxillary first molar
U6r	Mesiobuccal root apex of the maxillary first molar
U5c	Buccal cusp tip of the maxillary second premolar
U5r	Root apex of the maxillary second premolar
U3c	Cusp tip of the maxillary canine
U3r	Root apex of the maxillary canine
U2c	Midpoint of maxillary lateral incisor’s incisal edge
U2r	Root apex of the maxillary lateral incisor
U1c	Midpoint of maxillary central incisor’s incisal edge
U1r	Root apex of the maxillary central incisor

### Outcome measurements

The outcome measurement constituted changes in the 3-D positions of maxillary teeth. The 3-D crown and root movements of the maxillary teeth were measured separately. The inclination of the maxillary teeth was also analyzed, including the labiolingual inclination of the maxillary anterior teeth (central incisors, lateral incisors, and canines), the buccolingual inclination of maxillary posterior teeth (second premolars and first molars), as well as the mesiodistal inclination of the maxillary canines, second premolars, and first molars.

### Statistical analysis

To assess measurement reliability, 10 of the included patients were randomly selected, the landmarks were positioned, and the measurements were performed twice with a 2-week interval between measurements. The intra-class correlation coefficient of all measurements was ≥ 0.914, indicating good reliability. The method error was measured using the Dahlberg formula; it ranged from 0.14 to 0.31 mm for linear measurements, and from 0.12° to 0.32° for angular measurements. The normalities of measurement distributions were assessed using the Shapiro–Wilk test. For normally distributed measurements, repeated measures analysis of variance with post hoc LSD test was used to evaluate differences among groups. The paired t tests were used to analyze dental changes in each group from T0 to T1. When measurements were not normally distributed, the Wilcoxon signed-rank test was used. *P* < 0.05 was considered indicative of statistical significance. The effect sizes were measured using Cohen’s standardized mean difference. All analyses were performed using SPSS software (ver. 20.0, IBM Corp., Armonk, NY, USA).

## Results

### Participant flow

**Fig. 3:**
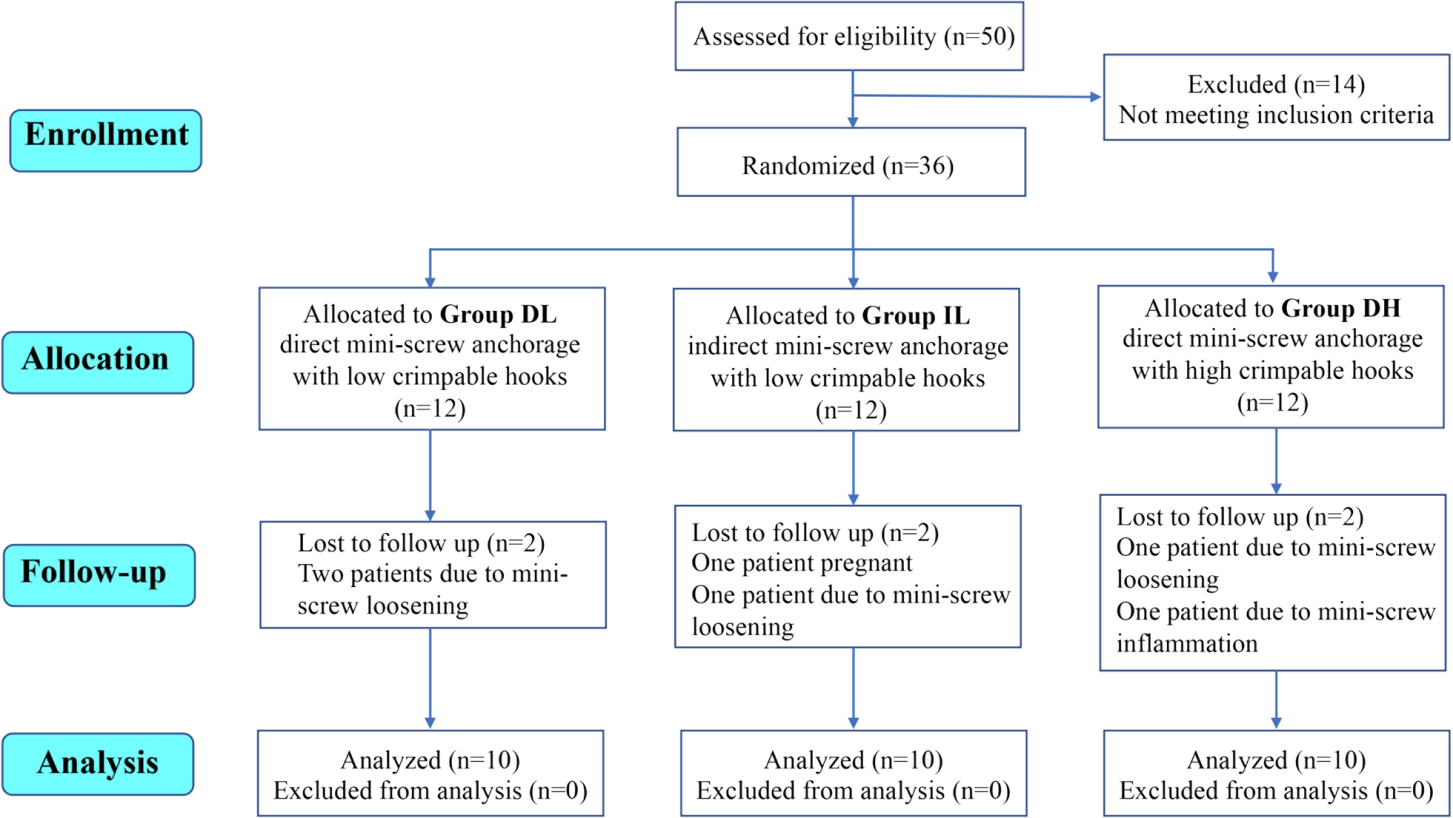
CONSORT flow diagram

In our study, a total of 50 patients were eligible for participation. Thirty-six patients who met the inclusion criteria were enrolled and randomly allocated into three groups. Among these patients, six were lost to follow-up during the space closure duration: two in the DL group because of miniscrew loosening, two in the IL group because of either miniscrew loosening or pregnancy, and two in the DH group because of miniscrew loosening and inflammation. Finally, 30 patients were included in our study: 10 in the DL group, 10 in the IL group, and 10 in the DH group. The CONSORT flow diagram is shown in Fig. 3.

### Baseline measurements

Demographic and baseline data of patients in the three groups are shown in Table 2. There were no significant differences in age, sex distribution, dental classification, extraction plan, extraction space, or cephalometric measurements at T0 among the three groups. However, space closure duration was significantly longer for patients in the DH group than for patients in the DL or IL groups.

**Table 2:** Demographic information of including patients

Measurements	Group DL (*n* = 10)	Group IL (*n* = 10)	Group DH (*n* = 10)	*P*
	Mean ± SD	Mean ± SD	Mean ± SD	
Age, years	22.50 ± 4.70	22.90 ± 3.54	22.40 ± 4.93	0.337
Sex (male/female)	2/8	1/9	0/10	0.217
Angle classification (class I/class II)	6/4	6/4	5/5	0.779
Extraction plan (14, 24, 34, 44 / 14, 24, 35, 45)	8/2	9/1	7/3	0.535
Spacing (mm)	6.39 ± 0.64	6.43 ± 0.70	6.42 ± 0.80	0.988
Spacing closure duration (months)	9.67 ± 2.18	8.50 ± 2.03	11.61 ± 2.06	< 0.001^**^
Overjet T0 (mm)	4.22 ± 0.55	4.05 ± 0.97	4.17 ± 0.73	0.880
SNA T0 (°)	80.57 ± 3.86	82.22 ± 2.98	82.54 ± 1.97	0.314
SNB T0 (°)	77.00 ± 4.01	79.35 ± 2.80	78.69 ± 1.93	0.222
ANB T0 (°)	3.56 ± 1.62	2.88 ± 0.99	3.90 ± 1.18	0.216
SN-MP T0 (°)	37.64 ± 5.93	36.39 ± 6.21	37.97 ± 5.19	0.735
U1-SN T0 (°)	110.55 ± 2.92	113.89 ± 4.52	111.23 ± 4.52	0.069

*P < 0.05, ^**^P < 0.01

### Outcomes

The 3-D changes in maxillary teeth in three groups are shown in Table 3. Positive values indicate lingual movement, extrusion, or distal movement of the crown and root, as well as lingual inclination and distal inclination of the tooth axis. Negative values indicate buccal movement, intrusion, or mesial movement of the crown and root, as well as buccal inclination, mesial inclination, or labial inclination of the tooth axis. As shown in Table 4, the 3-D changes of maxillary first molar after en masse retraction were significantly different among groups.

**Table 3:** Three-dimensional changes of maxillary teeth in three miniscrew anchorage groups

Measurements	Group DL (*n* = 20)		Group IL (*n* = 20)		Group DH (*n* = 20)	
	T0–T1 Mean (95% CI)	*P*	T0–T1 Mean (95% CI)	*P*	T0–T1 Mean (95% CI)	*P*
*Buccolingual/labiolingual position (mm)*						
U6c	− 0.65 (− 0.92 ~ − 0.37)	< 0.001^**^	0.75 (0.28 ~ 1.21)	0.004^**^	− 0.02 (− 0.45 ~ 0.41)	0.904
U6r	1.46 (0.87 ~ 2.04)	< 0.001^**^	0.38 (− 0.28 ~ 1.04)	0.253	0.16 (− 0.36 ~ 0.67)	0.515
U5c	− 0.16 (− 0.52 ~ 0.20)	0.478	0.88 (0.44 ~ 1.31)	< 0.001^**^	0.46 (0.12 ~ 0.79)	0.018^*^
U5r	0.71 (0.28 ~ 1.13)	0.006^**^	0.37 (− 0.15 ~ 0.88)	0.081	0.02 (− 0.34 ~ 0.38)	0.907
U3c	− 0.46 (− 1.18 ~ 0.27)	0.217	0.35 (− 0.32 ~ 1.01)	0.299	0.14 (− 0.38 ~ 0.65)	0.588
U3r	− 1.37(− 2.01 ~ − 0.73)	< 0.001^**^	− 0.44 (− 1.20 ~ 0.32)	0.214	− 1.61 (− 2.35 ~ − 0.87)	< 0.001^*^
U2c	5.09 (4.55 ~ 5.63)	< 0.001^**^	4.60 (3.81 ~ 5.38)	< 0.001^**^	5.11 (4.54 ~ 5.65)	< 0.001^*^
U2r	1.73 (1.03 ~ 2.43)	< 0.001^**^	1.69 (1.25 ~ 2.12)	< 0.001^**^	2.55 (2.05 ~ 3.04)	< 0.001^*^
U1c	5.63 (5.27 ~ 5.98)	< 0.001^**^	4.95 (4.65 ~ 5.25)	< 0.001^**^	5.05 (4.62 ~ 5.47)	< 0.001*
U1r	1.75 (1.14 ~ 2.36)	< 0.001^**^	1.69 (1.10 ~ 2.28)	< 0.001^**^	2.88 (2.25 ~ 3.51)	< 0.001*
*Vertical position (mm)*						
U6c	− 1.34 (− 1.60 ~ − 1.08)	< 0.001^**^	− 0.17 (− 0.50 ~ 0.17)	0.265	− 0.50 (− 0.84 ~ − 0.15)	0.007^**^
U6r	− 1.15 (− 1.61 ~ − 0.68)	< 0.001^**^	0.03 (− 0.29 ~ 0.34)	0.824	− 0.25 (− 0.69 ~ 0.19)	0.266
U5c	− 0.21 (− 0.69 ~ 0.28)	0.389	− 0.40 (− 0.87 ~ 0.08)	0.109	− 0.26 (− 0.51 ~ − 0.01)	0.042^*^
U5r	− 0.30 (− 0.71 ~ 0.12)	0.153	− 0.26 (− 0.62 ~ 0.10)	0.126	− 0.30 (− 0.58 ~ − 0.02)	0.036^*^
U3c	− 0.23 (− 0.58 ~ 0.13)	0.181	− 0.41 (− 0.99 ~ 0.17)	0.166	− 0.57 (− 0.99 ~ − 0.14)	0.011^*^
U3r	− 0.07 (− 0.35 ~ 0.22)	0.584	− 0.52 (− 1.04 ~ 0.01)	0.049^*^	− 0.36 (− 1.30 ~ 0.59)	0.450
U2c	− 0.31 (− 0.67 ~ 0.05)	0.088	− 0.41 (− 0.96 ~ 0.15)	0.138	− 1.08 (− 1.57 ~ − 0.59)	< 0.001^**^
U2r	0.14 (− 0.34 ~ 0.62)	0.527	− 0.72 (− 1.13 ~ − 0.31)	< 0.001^**^	− 1.16 (− 1.63 ~ − 0.68)	< 0.001^**^
U1c	0.09 (− 0.38 ~ 0.55)	0.772	− 0.39 (− 0.95 ~ 0.17)	0.158	− 1.29 (− 1.85 ~ − 0.72)	< 0.001^**^
U1r	0.47 (− 0.27 ~ 1.20)	0.192	− 0.99 (− 1.50 ~ − 0.48)	0.001*	− 0.97 (− 1.38 ~ − 0.56)	< 0.001^*^
*Mesiodistal position (mm)*						
U6c	0.44 (0.15 ~ 0.73)	0.004^**^	− 0.40 (− 0.83 ~ 0.03)	0.044^*^	0.62 (0.28 ~ 0.96)	0.002^**^
U6r	− 1.07 (− 1.49 ~ − 0.65)	< 0.001^**^	− 0.49 (− 1.00 ~ 0.03)	0.040^*^	0.11 (− 0.32 ~ 0.53)	0.708
U5c	0.10 (− 0.35 ~ 0.55)	0.683	− 0.59 (− 1.07 ~ − 0.11)	0.021^*^	0.22 (− 0.74 ~ 1.18)	0.624
U5r	0.22 (− 0.34 ~ 0.78)	0.400	− 0.69 (− 1.08 ~ − 0.30)	0.002^**^	0.02 (− 0.32 ~ 0.35)	0.825
U3c	4.41 (3.56 ~ 5.25)	< 0.001^**^	3.86 (2.97 ~ 4.75)	< 0.001^**^	5.14 (4.11 ~ 6.17)	< 0.001^**^
U3r	2.17 (1.38 ~ 2.95)	< 0.001^**^	2.59 (1.87 ~ 3.30)	< 0.001^**^	3.98 (3.06 ~ 4.89)	< 0.001^**^
U2c	− 0.05 (− 0.72 ~ 0.63)	0.890	0.15 (− 0.35 ~ 0.65)	0.563	0.16 (− 0.23 ~ 0.54)	0.472
U2r	− 1.12 (− 1.52 ~ − 0.71)	< 0.001^**^	− 0.66 (− 1.13 ~ − 0.19)	0.008^**^	− 1.57 (− 2.07 ~ − 1.06)	< 0.001^**^
U1c	− 0.09 (− 0.61 ~ 0.44)	0.753	− 0.04 (− 0.42 ~ 0.35)	0.850	− 0.21 (− 0.50 ~ 0.08)	0.132
U1r	− 0.41 (− 0.79 ~ − 0.03)	0.031^*^	− 0.15 (− 0.40 ~ 0.10)	0.210	− 0.31 (− 0.66 ~ 0.04)	0.080
*Buccolingual/labiolingual inclination (°)*						
U6	− 6.92 (− 8.67 ~ − 5.13)	< 0.001^**^	1.38 (− 1.50 ~ 4.25)	0.328	− 0.63 (− 3.01 ~ 1.76)	0.588
U5	− 2.26 (− 3.41 ~ − 1.10)	0.001^**^	1.27 (− 0.83 ~ 3.36)	0.220	1.30 (0.16 ~ 2.44)	0.027^*^
U3	2.86 (0.09 ~ 5.62)	0.044^*^	2.13 (− 0.02 ~ 4.28)	0.054	4.56 (2.80 ~ 6.32)	< 0.001^**^
U2	9.07 (7.05 ~ 11.08)	< 0.001^**^	7.91 (5.70 ~ 10.11)	< 0.001^**^	6.88 (5.31 ~ 8.44)	< 0.001^**^
U1	9.00 (7.14 ~ 10.84)	< 0.001^**^	8.39 (6.65 ~ 10.12)	< 0.001^**^	5.04 (2.82 ~ 7.26)	< 0.001^**^
*Mesiodistal inclination (°)*						
U6	4.90 (3.75 ~ 6.04)	< 0.001^**^	0.30 (− 1.78 ~ 2.37)	0.769	1.59 (0.26 ~ 2.91)	0.022^*^
U5	− 0.04 (− 2.20 ~ 2.12)	0.981	0.32 (− 1.18 ~ 1.81)	0.676	− 1.52 (− 9.20 ~ 6.17)	0.685
U3	5.12 (3.35 ~ 6.88)	< 0.001^**^	3.20 (0.94 ~ 5.46)	0.008^**^	2.56 (0.45 ~ 4.67)	0.020^*^

*P < 0.05, ^**^P < 0.01

**Table 4:** Comparison of three-dimensional changes of maxillary teeth among groups

Measurements	DL vs. IL vs. DH			DL vs. IL			DL vs. DH			IL vs. DH		
	F	*P*	Effect size^a^	Mean difference (95% CI)	*P*	Effect size^b^	Mean difference (95% CI)	*P*	Effect size^b^	Mean difference (95% CI)	*P*	Effect size^b^
*Buccolingual/labiolingual position (mm)*												
U6c	13.52	< 0.001^**^	0.69	− 1.40 (− 1.93 ~ − 0.86)	< 0.001^**^	1.71	− 0.63 (− 1.16 ~ − 0.09)	0.024^*^	0.81	0.77 (0.23 ~ 1.31)	0.006^**^	0.81
U6r	6.10	0.004^**^	0.46	1.08 (0.28 ~ 1.87)	0.009^**^	0.81	1.30 (0.50 ~ 2.10)	0.002^**^	1.11	0.23 (− 0.57 ~ 1.02)	0.574	0.18
U5c	8.118	0.001^**^	0.53	− 1.04 (− 1.55 ~ − 0.52)	< 0.001^**^	1.20	− 0.62 (− 1.13 ~ − 0.10)	0.021^*^	0.82	0.42 (− 0.10 ~ 0.94)	0.110	0.50
U5r	2.695	0.076	0.31	0.34 (− 0.25 ~ 0.93)	0.254	0.34	0.69 (0.09 ~ 1.28)	0.024^*^	0.81	0.35 (− 0.25 ~ 0.94)	0.247	0.37
U3c	1.848	0.167	0.27	− 0.80 (− 1.66 ~ 0.06)	0.069	0.30	− 0.59 (− 1.45 ~ 0.27)	0.177	0.44	0.21 (− 0.65 ~ 1.07)	0.628	0.17
U3r	3.288	0.045^*^	0.46	− 0.93 (− 1.90 ~ 0.04)	0.059	0.26	0.24 (− 0.73 ~ 1.21)	0.620	0.16	1.17 (0.20 ~ 2.14)	0.018^*^	0.73
U2c	0.896	0.414	0.18	0.50 (− 0.36 ~ 1.36)	0.251	0.35	0.01 (− 0.86 ~ 0.87)	0.989	0.00	− 0.50 (− 1.37 ~ 0.36)	0.252	0.34
U2r	3.327	0.043^*^	0.34	0.05 (− 0.71 ~ 0.80)	0.905	0.04	− 0.82 (− 1.57 ~ − 0.06)	0.034^*^	0.63	− 0.86 (− 1.61 ~ − 0.11)	0.026^*^	0.86
U1c	4.374	0.017^*^	0.39	0.68 (0.18 ~ 1.17)	0.008^**^	0.96	0.58 (0.09 ~ 1.07)	0.022^*^	0.69	− 0.10 (− 0.59 ~ 0.40)	0.702	0.12
U1r	5.264	0.008^*^	0.43	0.06 (− 0.77 ~ 0.89)	0.885	0.05	− 1.13 (− 1.96 ~ − 0.30)	0.008^*^	0.85	− 1.19 (− 2.02 ~ − 0.36)	0.006^*^	0.91
*Vertical position (mm)*												
U6c	16.11	< 0.001^**^	0.75	− 1.18 (− 1.60 ~ − 0.75)	< 0.001^**^	1.84	− 0.85 (− 1.27 ~ − 0.42)	< 0.001^**^	1.29	0.33 (− 0.10 ~ 0.76)	0.128	0.45
U6r	9.54	< 0.001^**^	0.58	− 1.17 (− 1.73 ~ − 0.61)	< 0.001^**^	1.37	− 0.90 (− 1.46 ~ − 0.33)	0.002^**^	0.92	0.28 (− 0.29 ~ 0.84)	0.330	0.33
U5c	0.243	0.785	0.53	0.19 (− 0.37 ~ 0.75)	0.501	0.19	0.06 (− 0.51 ~ 0.62)	0.845	0.07	− 0.14 (− 0.70 ~ 0.43)	0.632	0.17
U5r	0.017	0.984	0.31	− 0.04 (− 0.52 ~ 0.45)	0.885	0.04	0.01 (− 0.48 ~ 0.49)	0.983	0.01	0.04 (− 0.44 ~ 0.52)	0.868	0.06
U3c	0.589	0.558	0.14	0.19 (− 0.44 ~ 0.81)	0.558	0.18	0.34 (− 0.29 ~ 0.97)	0.283	0.41	0.16 (− 0.47 ~ 0.78)	0.623	0.14
U3r	0.553	0.578	0.14	0.45 (− 0.42 ~ 1.32)	0.304	0.50	0.29 (− 0.58 ~ 1.16)	0.506	0.20	− 0.16 (− 1.03 ~ 0.71)	0.714	0.10
U2c	3.420	0.040^*^	0.35	0.10 (− 0.55 ~ 0.74)	0.768	0.10	0.77 (0.13 ~ 1.41)	0.020^*^	0.84	0.68 (0.03 ~ 1.32)	0.040^*^	0.60
U2r	9.201	< 0.001^**^	0.57	0.86 (0.24 ~ 1.48)	0.007^**^	0.91	1.30 (0.68 ~ 1.91)	< 0.001^**^	1.27	0.44 (− 0.18 ~ 1.05)	0.162	0.46
U1c	7.474	0.001^**^	0.51	0.48 (− 0.25 ~ 1.20)	0.192	0.43	1.37 (0.65 ~ 2.09)	< 0.001^**^	1.24	0.90 (0.17 ~ 1.62)	0.016^*^	0.74
U1r	9.397	< 0.001^**^	0.57	1.46 (0.68 ~ 2.23)	< 0.001^**^	1.08	1.44 (0.66 ~ 2.21)	< 0.001^**^	1.13	− 0.02 (− 0.79 ~ 0.75)	0.959	0.02
*Mesiodistal position (mm)*												
U6c	10.30	< 0.001^**^	0.60	0.84 (0.36 ~ 1.32)	0.001^**^	1.08	− 0.18 (− 0.66 ~ 0.30)	0.456	0.27	− 1.02 (− 1.50 ~ − 0.54)	< 0.001^**^	1.24
U6r	7.30	0.002^**^	0.51	− 0.59 (− 1.20 ~ 0.03)	0.062	0.58	− 1.18 (− 1.79 ~ − 0.56)	< 0.001^**^	1.30	− 0.59 (− 1.21 ~ 0.03)	0.060	0.59
U5c	1.852	0.166	0.26	0.69 (− 0.22 ~ 1.60)	0.134	0.69	− 0.12 (− 1.03 ~ 0.79)	0.793	0.08	− 0.81(− 1.72 ~ 0.10)	0.080	0.50
U5r	5.163	0.009^**^	0.43	0.91 (0.32 ~ 1.50)	0.003^**^	0.88	0.21 (− 0.39 ~ − 0.80)	0.493	0.21	− 0.71 (− 1.30 ~ − 0.11)	0.021^*^	0.91
U3c	2.129	0.128	0.26	0.55 (− 0.70 ~ 1.80)	0.382	0.54	− 0.74 (− 1.99 ~ 0.52)	0.244	0.37	− 1.29 (− 2.54 ~ − 0.03)	0.044^*^	0.62
U3r	6.020	0.004^**^	0.34	− 0.42 (− 1.51 ~ 0.67)	0.445	0.62	− 1.81 (− 2.90 ~ − 0.72)	0.002^**^	1.00	− 1.39 (− 2.48 ~ − 0.30)	0.014^*^	0.80
U2c	0.199	0.820	0.08	− 0.20 (− 0.92 ~ 0.53)	0.592	0.15	− 0.20 (− 0.92 ~ 0.52)	0.582	0.17	− 0.01 (− 0.73 ~ 0.72)	0.989	0.01
U2r	4.156	0.021^*^	0.38	− 0.46 (− 1.08 ~ 0.17)	0.153	0.48	0.45 (− 0.18 ~ 1.08)	0.157	0.46	0.91 (0.28 ~ 1.53)	0.006^**^	0.86
U1c	0.212	0.810	0.09	− 0.05 (− 0.60 ~ 0.50)	0.857	0.05	0.13 (− 0.43 ~ 0.68)	0.653	0.14	0.18 (− 0.38 ~ 0.73)	0.530	0.24
U1r	0.687	0.507	0.16	− 0.26 (− 0.71 ~ 0.19)	0.250	0.38	− 0.10 (− 0.55 ~ 0.35)	0.657	0.13	0.16 (− 0.29 ~ 0.61)	0.477	0.25
*Buccolingual/labiolingual inclination (°)*												
U6	14.29	< 0.001^**^	0.71	− 8.27 (− 11.51 ~ − 5.04)	< 0.001^**^	1.62	− 6.28 (− 9.51 ~ 3.04)	< 0.001^**^	1.40	2.00 (− 1.23 ~ 15.23)	0.221	0.35
U5	7.808	0.001^**^	0.52	− 3.52 (− 5.59 ~ − 1.45)	0.001^**^	0.98	− 3.56 (− 5.62 ~ − 1.49)	0.001^**^	1.45	− 0.04 (− 2.10 ~ 2.03)	0.973	0.01
U3	1.328	0.273	0.22	0.73 (− 2.34 ~ 3.79)	0.638	0.14	− 1.71 (− 4.77 ~ 1.36)	0.270	0.34	− 2.43 (− 5.50 ~ 0.64)	0.118	0.58
U2	1.388	0.258	0.22	1.16 (− 1.47 ~ 3.79)	0.382	0.26	2.19 (− 0.44 ~ 4.82)	0.101	0.57	1.03 (− 1.60 ~ 3.66)	0.437	0.25
U1	5.226	0.008^*^	0.43	0.61 (− 2.03 ~ 3.24)	0.648	0.16	3.95 (1.31 ~ 6.59)	0.004^**^	0.90	3.35 (0.71 ~ 5.98)	0.014^*^	0.79
*Mesiodistal inclination (°)*												
U6	10.04	< 0.001^**^	0.59	4.60 (2.48 ~ 6.72)	< 0.001^**^	1.29	3.31 (1.19 ~ 5.43)	0.003^**^	1.25	− 1.29 (− 3.41 ~ 0.83)	0.228	0.35
U5	0.187	0.830	0.08	− 0.36 (− 6.70 ~ 6.00)	0.911	0.09	1.48 (− 4.87 ~ 7.82)	0.643	0.12	1.83 (− 4.52 ~ 8.18)	0.566	0.16
U3	1.834	0.169	0.25	1.92 (− 0.86 ~ 4.69)	0.173	0.44	2.56 (− 0.22 ~ 5.33)	0.071	0.62	0.64 (− 2.14 ~ 3.42)	0.647	0.14

^a^Effect sizes (small = 0.10, moderate = 0.25, large ≥ 0.40) were measured using Cohen’s f

^b^Effect sizes (small = 0.20, moderate = 0.50, large ≥ 0.80) were measured using Cohen’s d

*P < 0.05, ^**^P < 0.01

A significant intrusion (U6c: − 1.34 mm; 95% CI, − 1.60 mm, 1.08 mm) and buccal (− 6.92°; 95% CI, − 8.67°, − 5.13°) and distal (4.90°; 95% CI, 3.75°, 6.04°) inclination of the maxillary first molars were observed in the DL group, compared to the other two groups. In addition, mesial movement (U6c: − 0.40 mm; 95% CI, − 0.83 mm, − 0.03 mm) of the maxillary first molars was found in the IL group, while the DL (U6c: 0.44 mm; 95% CI, 0.15 mm, 0.73 mm) and DH (U6c: 0.62 mm; 95% CI, 0.28 mm, 0.96 mm) groups exhibited distal movement. The second premolars were buccally inclined (− 2.26°; 95% CI, − 3.41°, − 1.10°) in the DL group, whereas they were lingually inclined in the IL (1.27°; 95% CI, − 0.83°, 3.36°) and DH (1.30°; 95% CI, 0.16°, 2.44°) groups. The differences of vertical and mesiodistal change of second premolars among groups were not significant.

As for maxillary anterior teeth, the maxillary canines were lingually and distally inclined in all three groups, which the changes of labiolingual and mesiodistal inclination among groups were not significantly different. The distal movement of canine root apexes (3.98 mm; 95% CI, 3.06 mm, 4.89 mm) was significantly greater in the DH group than in the other two groups. In the DH group, the lingual inclination changes of maxillary central incisor (5.04°; 95% CI, 2.82°, 7.26°) were significantly lower, and the lingual movement of the incisor root apexes (2.88 mm; 95% CI, 2.25 mm, 3.51 mm) was significantly greater, which is indicative of good lingual root torque control of the maxillary anterior teeth. Besides, the intrusion of maxillary central incisor (− 1.29 mm; 95% CI, − 1.85 mm, − 0.72 mm) and lateral incisor (− 1.08 mm; 95% CI, − 1.57 mm, − 0.59 mm) were significant in DH group, while the other two groups exhibited no significant vertical changes.

### Harms

Six miniscrews in five patients were loosened and removed. No serious harm was observed during extraction space closure.

## Discussion

The miniscrew has been widely used to enhance maxillary anchorage in patients with maxillary dentoalveolar protrusion. Thus far, many studies have reported comparisons of anchorage capacity between miniscrews and traditional anchorages [3, 6, 12]. Given the controversial effects of different sliding mechanics using miniscrews during space closure, it is essential to conduct a prospective clinical trial to thoroughly investigate their effects on tooth movement. CBCT is considered an accurate and efficient tool for evaluating tooth movement during orthodontic treatment [18, 19]. Therefore, we used 3-D superimpositions of pre-retraction and post-retraction CBCT images to explore maxillary tooth movements in three common miniscrew anchorage systems.

In our study, distal movement of the maxillary first molars was observed in the DL (0.44 mm; 95% CI, 0.15 mm, 0.73 mm) and DH (0.62 mm; 95% CI, 0.28 mm, 0.96 mm) groups, while the maxillary first molars in the IL group moved mesially (− 0.40 mm; 95% CI, − 0.83 mm, 0.03 mm). In the two direct miniscrew anchorage groups, the distal movements of the first molars could be attributed to the frictional force between the arch wire and bracket. Our findings are consistent with previous reports by Monga et al. and Upadhyay et al. [10, 20]. We also observed significant buccal and distal inclination and intrusion of the maxillary first molars in the DL group; such results have also been previously reported [7, 8, 21]. These vertical and transverse changes in the first molars were caused by vertical and transverse components of the retraction force.

The miniscrews were located in buccal alveolar bone regions at a high position; the retraction force from miniscrew to low anterior hook is not parallel to the posterior segment either vertically or transversally. While the retraction force was more parallel to the occlusal plane in the IL and DH groups, the sliding mechanics in the IL and DH groups were similar to the sliding mechanics with conventional anchorage. Thus, vertical and transverse anchorage loss should be considered when miniscrews are used in combination with low crimpable hooks. To stabilize the maxillary first molars, the Nance appliance or transpalatal arch could be used as adjuncts; the second molars should be banded early. In growing patients, mesial movement of the maxillary first molars reportedly could occur, partially because of natural growth [22]. In our study, all included patients were adults; thus, the influence of natural growth could be disregarded.

The 3-D changes in maxillary second premolars during en masse retraction were less reported in previous studies. The maxillary second premolar was located in the middle of maxillary dental arch. In the sliding mechanism system, the force applied to the maxillary second premolars was mainly frictional force; the vertical and transverse vectors were small. Thus, the 3-D positions of the second premolars were more stable than the 3-D positions of the other maxillary teeth. Notably, although the miniscrews were tightened to the second premolar brackets in the IL group, the crown of the second premolars moved mesially (− 0.59 mm; 95% CI, − 1.07 mm, − 0.11 mm) and intruded (− 0.40 mm; 95% CI, − 0.87 mm, 0.08 mm).

In all three miniscrew groups, the canines inclined lingually and distally under the retraction force. A possible explanation for this is that the path of canine movement was not parallel to the retraction force because the canines are located at the corners of the maxillary dental arch. The morphology of the canine root is long and bulbous, which makes bodily movement particularly difficult [23]. In our study, significant buccal movement of the canine roots occurred in direct miniscrew groups (DL and DH groups). Some CBCT studies have indicated that canine roots are usually positioned close to the maxillary cortical bone [24, 25]. In a previous systematic review, we found that maxillary canines had a high risk of alveolar bone dehiscence and fenestration in extraction patients [26]. Therefore, lingual root torque control of the canines should be enhanced in extraction cases. A high-torque bracket can be used, or additional lingual root torque can be performed.

Torque control of the maxillary anterior teeth is critical to the success of extraction treatment. In a traditional sliding mechanism, it is commonly known that the maxillary incisors incline lingually under retraction forces. Previous studies have reported that the center of resistance of the anterior teeth is located 13.5 mm apically and 14 mm posteriorly to the central edge of the maxillary central incisors [8, 27]. In our study, the retraction force from the miniscrews to the high crimpable hooks was close to the center of resistance of the anterior segment. Thus, compared to the significant lingual inclination of the maxillary incisors in the DL and IL groups, good lingual root torque control of the maxillary incisors was observed in the DH group. Lee et al. also found a bodily movement of maxillary anterior teeth during en masse retraction with miniscrews and high crimpable hooks [28]. Therefore, high crimpable hooks enable the achievement of better anterior torque control during en masse retraction [29]. However, the duration of space closure was significantly longer for patients in the DH group, presumably because of increased anterior torque control.

Lingual inclination of the maxillary incisors can lead to an extrusion effect. Cho et al. reported 2 mm of extrusion of the maxillary incisors with conventional anchorages after retraction [30]. The degree of vertical change in the maxillary anterior teeth in miniscrew anchorage patients remains controversial. After the retraction of anterior teeth with direct miniscrew anchorages and low crimpable hooks, Upadhyay et al. found significant intrusion of the maxillary incisors [11]. In contrast, Lee et al. reported no significant vertical changes in maxillary incisor positions [6]. In our study, the vertical position of the maxillary incisors was stable in the DL group, presumably because the low crimpable hooks increased the vertical retraction force; this could counteract the slight extrusion produced by lingual inclination of the anterior teeth and thus maintain the vertical position of maxillary incisors. When retracting anterior teeth with direct miniscrew anchorages and high crimpable hooks, intrusion of the maxillary incisors by 1.24 mm occurred because of the increased anterior torque control. Consistent with our findings, Salma et al. reported intrusion of 1.53 mm after space closure using miniscrews and high crimpable hooks [31].

Orthodontists should be aware of the 3-D maxillary tooth movement in different miniscrew anchorage systems. This way, the target tooth movement could be achieved by selecting the appropriate anchorage system or using adjunctive appliances. Notably, some unwanted tooth movement in one group may be desired in another. For instance, the intrusion of maxillary incisors in DH group is detrimental in open bite cases but beneficial for patients with a deep bite.

### Limitations

This prospective study was designed as a randomized controlled trial. Firstly, the nature of the intervention impeded double-blinding. In this study, the patients were randomly selected and the examiner was blinded to the group assignments when measuring outcome variables and performing statistical analyses. Secondly, the sample size was relatively small. In our study, two sides of the upper dentition in each patient were analyzed separately to double the sample size. Most of our including patients were females. Although the sex distribution among groups was not significantly different, the sex distribution within groups was different. Hence, the results may not be generalizable to a larger population. Thirdly, the maxillary second molars were not included during en masse retraction. The sliding mechanics might have differed if the maxillary second molars had been included; future studies should be designed to analyze its 3-D movements.

## Conclusion

1. For extraction patients with a maxillary dentoalveolar protrusion, the maxillary first molars were significantly buccally and distally inclined and intruded using direct miniscrew anchorages with low crimpable hooks.
2. In all three miniscrew groups, the maxillary canines were lingually and distally inclined under retraction force. Lingual root torque control of canines should be enhanced to prevent dehiscence and fenestration.
3. Good lingual root torque control of maxillary incisors was observed in patients using direct miniscrew anchorages with high crimpable hooks.
4. The vertical position of the maxillary incisors was stable in patients using direct miniscrew anchorages with low crimpable hooks, while these teeth were slightly intruded in patients using direct miniscrew anchorages with high crimpable hooks.

## Data Availability

The datasets used and/or analyzed during the current study are available from the corresponding author on reasonable request.
